# Accuracy of 3-dimensional echocardiography in measuring right ventricular volumes and ejection fraction: a systematic review and meta-analysis

**DOI:** 10.1186/s44156-026-00102-w

**Published:** 2026-01-29

**Authors:** Mostafa Rafea, Ramy Kishk, Ramy Salmoun

**Affiliations:** 1 London North West Healthcare NHS Trust, Harrow, UK; 2 Royal Free Hospital, London, UK

**Keywords:** Right ventricular ejection fraction, Right ventricular end-diastolic volume (RVEDV), Right ventricular end-systolic volume (RVESV), Cardiac magnetic resonance, Right ventricular volumes, Three-dimensional echocardiography

## Abstract

**Background and Aims:**

The complex architecture of the right ventricle presents a significant challenge in determining its functional status. Three-dimensional echocardiography (3DE) is growing in prevalence as a faster and more convenient tool to evaluate Right Ventricular (RV) function. However, its accuracy and concordance with cardiac magnetic resonance imaging (CMR) is yet to be determined. In this study, we conducted a comprehensive systematic review and meta-analysis to evaluate the agreement of 3DE to CMR for RV functional assessment.

**Methods:**

We conducted a systematic literature review of PubMed, Embase, and the Cochrane Library through to December 2024. Studies were included if they directly compared 3DE and CMR measurements of RV function and volumes. Data synthesis was carried out with RevMan Web using a random-effects model.

**Results:**

Seventy-five studies were included in the analysis. 3DE systematically underestimated RV volumes compared to CMR, with significant differences for end-diastolic volume (SMD = −0.43, 95% CI: −0.55, −0.30) and end-systolic volume (SMD: −0.27, 95% CI: −0.39, −0.16). However, for RV ejection fraction, 3DE showed minimal underestimation (MD: −0.92%, 95% CI: −2.20, 0.36) with no statistically significant difference (p: 0.16).

**Conclusion:**

These findings support the potential clinical utility of 3DE for RV ejection fraction assessment while highlighting the continued importance of CMR for precise volume measurements.

**Supplementary information:**

The online version contains supplementary material available at 10.1186/s44156-026-00102-w.

## Introduction

Accurate evaluation of RV function and volumes is crucial in the management of multiple cardiovascular conditions [1, 2], being a strong prognostic indicator for pulmonary hypertension, left heart failure, and chronic obstructive pulmonary disease, among other conditions [3, 4]. Although 2D echocardiography remains the most commonly used technique in the assessment of RV function [5], it is challenging and is capable of measuring only surrogate parameters of RV function due to the complex ventricular geometry [6–10].

Cardiac magnetic resonance (CMR) is considered the reference method for quantitative RV assessment [2, 5]. However, it is often limited in availability, can be costly and may require anaesthesia particularly in paediatric population [11, 12]. Therefore, a reliable diagnostic tool that aligns closely with CMR is required.

With the significant advances in software and hardware, including new AI-based 3DE algorithms [5], 3D echocardiography has emerged in recent years as a promising imaging method and was proven to be reliable for RV function evaluation [12], but its use is still limited to research and specialised centres [12, 13].

A study by *Kitano et.al* (2023) has demonstrated the prognostic power of 3DE derived RVEF in prediction of adverse cardiovascular outcomes that is superior to LVEF [14], although prognostic value does not validate measurement accuracy. While earlier meta-analyses, such as that by Shimada et al. (2010) [15], provided important insights into the potential of 3DE, contemporary meta-analysis that take into account the recent advances in imaging technology are needed. Therefore, this meta-analysis aimed to comprehensively assess 3DE bias compared to MRI and investigate factors influencing systematic measurement differences.

## Methods

A comprehensive literature review for the studies that were published up to December 2024 was conducted across 3 databases (PubMed, Embase, and the Cochrane Library) following registration of our protocol on PROSPERO (registration number: CRD42024618897). This systematic review and meta-analysis were conducted and reported in accordance with the PRISMA guidelines. We used RAYYAN (a web-based platform) for screening of the studies. First, we screened title and abstract, followed by full texts. This was done by 2 independent reviewers according to the predefined criteria in the protocol.

We included all the studies that fulfilled the inclusion criteria using 3D echo as the index test for assessment of RVEF and volumes (RVEDV, RVESV) and cardiac MRI as the gold standard. We included both adult and paediatric populations as well as studies with different cardiac conditions, including heart failure, pulmonary hypertension, congenital heart diseases and postoperative patients. We excluded non-English studies, studies lacking quantitative measurements of RV function, systematic reviews and letters to the editor.

The QUADAS-2 tool (Quality Assessment of Diagnostic Accuracy Studies-2) was used to assess the quality of the studies. Data were extracted into a spreadsheet. Wan et al.’s (2014) method [16] was used to convert the measurements to mean and SD if the data was reported as median and IQR.

Meta-analysis was performed using RevMan Web (Cochrane software) with a random-effects model to account for study heterogeneity. We used standardised mean difference (SMD) with 95% confidence intervals for RV volumes since studies used different units (mL vs. mL/m^2^). However, mean difference (MD) was kept for EF as units did not differ across studies. Heterogeneity was assessed using the I^2^ statistic, with values > 50% indicating moderate to high heterogeneity.

Subgroup analysis was performed based on the following: age group (adults ≥18 years vs. paediatric < 18 years), tracking method (manual, semi-automatic, or fully automatic endocardial border detection), population type (normal hearts, diseased hearts, or mixed populations) and finally volume indexing (indexed vs. non indexed volumes) for clinical interpretation. Sensitivity analysis was performed by removing one study at a time to assess impact on overall results and heterogeneity.

Meta-regression analysis was performed using Open Meta-Analyst software to analyse the influence of year of publication, mean age, and sample size.

Pooled correlation coefficients were done using Jamovi software to assess the agreement between 3DE and CMR. Publication bias assessment was conducted using funnel plots and Egger’s test.

## Results

**Fig. 1:**
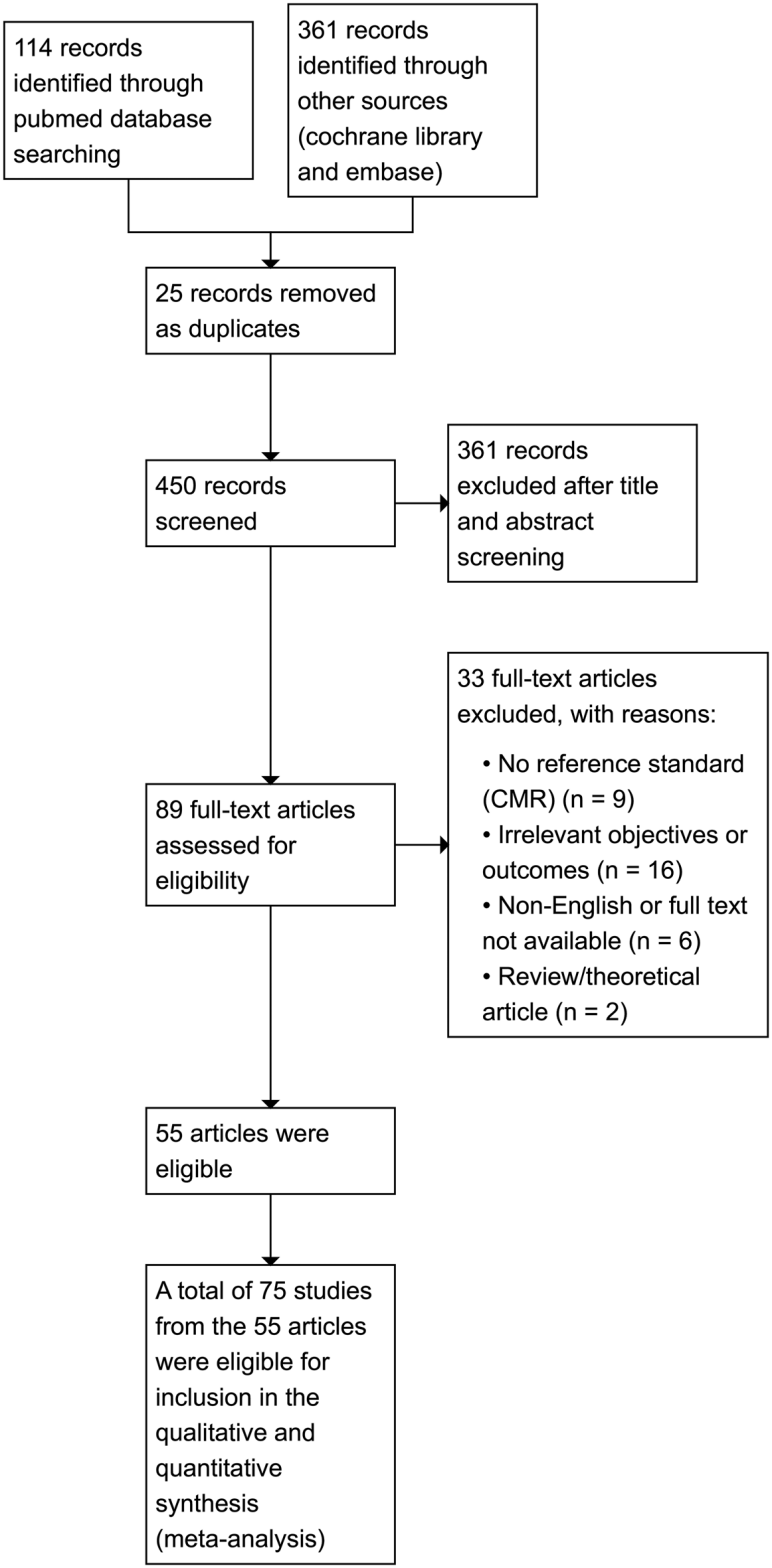
Flow_diagram

The search strategy yielded 55 articles, including 75 studies with a total of ~ 4300 patients (Fig. 1); baseline characteristics are in Table 1.

**Table 1:** Baseline characteristics

Study ID	Year	Population	Sample Size	Age (years)	3DE view and methods	Hardware	Software	Tracking of endocardium
Papavassiliou et al. 1998 [17]	1988	Status post tetralogy of Fallot repair (10 patients), Status post hypoplastic left heart syndrome repair (2 patients), Status post atrial septal defect repair (1 patient)	13	6.3 (1.4–12.9)	Apical or subcostal view, internal rotation at increments of 5–10 to gain cross-sectional images, then reconstructed	Hewlett-Packard Sonos 2500 with prototype internally rotating 5 MHz omniplane transducer	TomTec 3.0 workstation for 3D echocardiography processing	Manual
Angelini et al. 2005 (Deformable Model) [18]	2005	All had PHT: 8 with primary PH, 2 with secondary PH due to congenital defects	10	33 (6–63)	Standard apical 4-chamber view position	Matrix phased-array transducer (Volumetrics® technology, similar to Philips SONOS 7500 and iE33 systems).	Automated segmentation: 3D level-set deformable model (homogeneity-based active contour method).	Automatic
Angelini et al. 2005 (Manual Tracing) [18]	2005	All had PHT: 8 with primary PH, 2 with secondary PH due to congenital defects	9	33 (6–63)	Standard apical 4-chamber view position	Matrix phased-array transducer (Volumetrics® technology, similar to Philips SONOS 7500 and iE33 systems).	Manual tracing was performed using 3Dechotech© software (for RT3D-US) and Mass© v.5 (for MRI).	Manual
Kjaergaard et al. 2006 [19]	2006	Patients with prior inferior STEMI (*n* = 17), Patients with history of pulmonary embolism and persistent dyspnoea (*n* = 7), Normal subjects (*n* = 10)	34	IHD patients: 68 ± 10, PH patients: 68 ± 9, Normal: 70 ± 7	Apical views	Philips SONOS 7500. equipped with s3 and x3 transducers	TomTec 4D Echo-view	Not specified
Nesser et al. 2006 [20]	2006	DCM, CAD, valve disease, normal hearts (18 of them were in sinus rhythm and 2 in atrial fibrillation)	20	23–76	Parasternal, apical, or subcostal including low parasternal and a modified apical view, rotating transducer by 5degrees increments through 180 degrees to gain cross-sectional images, then reconstructed	VingMed, CFM800, Sonotron, Norway. And a standard 2.5 MHz transducer	TomTec Imaging Systems	Manual
Prakasa et al. 2006 [21]	2006	ARVD/C patients: 23, First-degree relatives without ARVD/C: 20, Idiopathic ventricular tachycardia without ARVD/C: 8, Healthy volunteers: 7	58	37 ± 11	Apical 4-chamber and modified apical 2-chamber views	Sonos 7500 or IE-33 (Philips Ultrasound)	TomTec	Manual
Gopal et al. 2007 (Disk Summation) [22]	2007	Healthy volunteers	71	56 ± 14.3	Modified apical view with DS method	A matrix-array transducer (24 MHz) (X4, Philips Imaging Systems)	TomTec Imaging Systems	Manual
Jenkins et al. 2007 [23]	2007	Patients with left ventricular wall motion abnormalities and suspected RV involvement after acute myocardial infarction	50	62 ± 11	Modified apical window, over 4 cardiac cycles, breath-hold ~10 seconds	Matrix array transducer (4 V transducer, Philips Sonos 7500)	4D Analysis (TomTec Gmbh)	Semi-automatic
Niemann et al. 2007 [24]	2007	Adults (Normal adults + HTN/CAD+ VSD): 14 patients, Paediatrics with congenital heart disease: 16 patients	30	Adults: 39 ± 22, Paediatrics: 9 ± 6	Apical and subcostal views	Philips 7500 system with matrix-array 3D transducer	TomTec prototype RV analysis program	Manual
Lu et al. 2008 [25]	2008	Healthy volunteers with no cardiovascular disease history	17	10.6 ± 2.8	Modified apical 4- and 2-chamber views	4-MHz X4 matrix-array transducer connected to a RT3DE system (Sonos 7500, Philips Medical Systems)	TomTec Imaging Systems	Manual
Iriart et al. 2009 [26]	2009	Healthy Volunteers: 14 (7 men, 7 women; mean age 32 ± 8 years), Repaired TOF Patients: 20 (11 men, 9 women; mean age 26 ± 10 years)	34	31 + 14	Modified apical view	4Z1c matrix-array transducer (1–4 MHz) and a Sequoia prototype ultrasound system (Siemens)	TomTec	Semi-automatic
Khoo et al. 2009 (AR method) [27]	2009	Patients with congenital heart disease	28	16.5 (12–25)	Modified apical window (with apical rotation method)	Real-time 3D using matrix-array transducers (X3-1 or X7-2 transducers, Philips ie33; Philips Medical Systems, Andover, MA).	EchoView version 5.4 and 4D RV Analysis (TomTec)	Manual
Khoo et al. 2009 (ABD method) [27]	2009	Patients with congenital heart disease	28	16.5 (12–25)	Modified apical window (with Automated Border Detection (ABD) method))	Real-time 3D using matrix-array transducers (X3-1 or X7-2 transducers, Philips ie33; Philips Medical Systems, Andover, MA).	EchoView version 5.4 and 4D RV Analysis (TomTec)	Semi-automatic
Khoo et al. 2009 (DS method) [27]	2009	Patients with congenital heart disease	28	16.5 (12–25)	Modified apical window (with DS method)	Real-time 3D using matrix-array transducers (X3-1 or X7-2 transducers, Philips ie33; Philips Medical Systems, Andover, MA).	EchoView version 5.4 and 4D RV Analysis (TomTec)	Manual tracing, 10 mm thick discs
Khoo et al. 2009 (MABD method) [27]	2009	Patients with congenital heart disease	28	16.5 (12–25)	modified apical window (with Manual Adjusted Border Detection method)	Real-time 3D using matrix-array transducers (X3-1 or X7-2 transducers, Philips ie33; Philips Medical Systems, Andover, MA).	EchoView version 5.4 and 4D RV Analysis (TomTec)	Semi-automatic
Grapsa et al. 2010 (Control group) [28]	2010	Healthy controls	20	39 ± 16.2 yr	Apical four-chamber views	GE Vivid 7 with X4 transducer, Frequency: 3–4 MHz, Frame rate: 16–24 frames/s	4D analysis, TomTec	Manual
Grapsa et al. 2010 (PAH) [28]	2010	Pulmonary arterial hypertension	60	42.8 ± 18.3 yr	Apical four-chamber views	GE Vivid 7 with X4 transducer, Frequency: 3–4 MHz, Frame rate: 16–24 frames/s	4D analysis, TomTec	Manual
Leibundgut et al. 2010 [29]	2010	Ischemic heart disease 19%, Idiopathic cardiomyopathy 11%, Myocarditis 25%, Arrhythmogenic right ventricular cardiomyopathy 13%, Storage disease 4%, Aortic dilatation 8%, Others 8%	88	50 ± 16	modified apical view	Philips iE33 with matrix-array X3-1 transducer	4D RV-Function CAP 1.1; TomTec Imaging Systems, RT3DE frames per cycle: 23 ± 5 (range 11–38)	Manual
Van Der Zwaan et al. 2010 (CHD) [30]	2010	Tetralogy of Fallot: 38, Aortic valve abnormality: 17, Pulmonary stenosis (with/without VSD): 12, Transposition of great arteries, atrial switch: 10, Transposition of great arteries, arterial switch: 9, Pulmonary atresia (with/without VSD): 4, Other conditions: 10	100	27 ± 10	Modified apical view	iE33 ultrasound system (Philips Medical Systems)	TomTec	Semi-automatic
Van Der Zwaan et al. 2010 (Healthy controls) [30]	2010	Healthy controls	41	27 ± 10	Modified apical view	iE33 ultrasound system (Philips Medical Systems)	TomTec	Semi-automatic
Crean et al. 2011 [31]	2011	CoA (*n* = 7), TOF (*n* = 14), TGA (*n* = 4)	25	CoA: 26 ± 6.8; TOF/TGA: 27 ± 5.2	modified standard views (most often apical 4 chamber)	Philips IE33	TomTec software	Semi-automated
Oomah et al. 2011 (after the marathon) [32]	2011	Healthy volunteers (nonelite volunteers participating in the 2009 Manitoba Half Marathon was performed)	15	32 ± 6	not specified	GE Vivid 7 (dedicated broadband, wide-angle, matrix-array transducer	(TomTec Imaging Systems	Not specified
Oomah et al. 2011 (Before the marathon) [32]	2011	Same as above	15	32 ± 6	not specified	GE Vivid 7 (dedicated broadband, wide-angle, matrix-array transducer	(TomTec Imaging Systems	Not specified
Van Der Zwaan et al. 2011 [33]	2011	Patients with tetralogy of Fallot, pulmonary stenosis, ventricular septal defect and transposition of the great arteries after an atrial switch	120	29 ± 11	Apical four-chamber view, short-axis view and coronal view in both phases	Philips Medical Systems	TomTec Imaging Systems	Semi-automatic
Ostenfeld et al. 2012 (Manually 3DE corrected data) [34]	2012	Ischemic heart disease: 37%, Congestive heart failure: 24%, Dilated cardiomyopathy: 10%, Valvular heart disease: 19%, Hypertrophic cardiomyopathy: 11%, Aortopathy: 6%, Ventricular arrhythmia: 19%, Peri-myocarditis: 5%	53	55 ± 16	Apical view (Acquisition: Full volume over 4–7 heart cycles, ECG-gated, breath-hold)	Sonos 7500 (*n* = 29) and iE33 (*n* = 33) with matrix array transducers	4D RV-Function (TomTec Imaging Systems)	Semi-automatic
Ostenfeld et al. 2012 (Uncorrected data) [34]	2012	Ischemic heart disease: 37%, Congestive heart failure: 24%, Dilated cardiomyopathy: 10%, Valvular heart disease: 19%, Hypertrophic cardiomyopathy: 11%, Aortopathy: 6%, Ventricular arrhythmia: 19%, Peri-myocarditis: 5%	53	55 ± 16	Same as above	Sonos 7500 (*n* = 29) and iE33 (*n* = 33) with matrix array transducers	4D RV-Function (TomTec Imaging Systems)	Semi-automatic
Fang et al. 2013 [35]	2013	Systolic heart failure (LVEF ≤50%) and thalassemia, Pulmonary hypertension was excluded	33	52 ± 23	Apical four chamber view	iE33, Phillips, United States	TomTec, RV function, Germany	Semi-automatic
Inaba et al. 2013 [36]	2013	IPAH: 5 patients, collagen vascular disease: 13 patients, congenital: 2 patients, portal hypertension: 2 patients, CTEPTH: 1 patient	23 patients had 3 DE and 10 had CMR	55.3 ± 17.2	Apical views	Philips Medical Systems	QLAB-3DQadv version 7.0 software (Philips Medical Systems)	Semi-automatic
Zhang et al. 2013 [37]	2013	No significant heart disease: 16 patients, Ischemic cardiomyopathy: 9 patients, Hypertension: 20 patients, Pulmonary heart disease: 14 patients	59	62 ± 14	Modified apical view	single-beat full-volume capture real-time three-dimensional echocardiography, using a special transducer (4Z1c) that has a matrix array with a maximum volume angle of 90°x 90°, Acuson SC2000 system; Siemens Medical Solutions	TomTec v1.2, TomTec Imaging Systems	Manual
Bell et al. 2014 [38]	2014	Hypoplastic left heart syndrome	28	0.37 (0.18–9.28)	Subcostal or apical projections	Philips IE33 ultrasound system	TomTec software	Semi-automatic
Kim et al. 2014 [39]	2014	Ischemic cardiomyopathy: 17 patients, non-ischemic cardiomyopathy: 10 patients	27	61 ± 14	Modified apical view	Philips iE33 with X3-1 matrix probe	TomTec, manual tracing + automatic volume modelling	Manual
Laser et al. 2014 (CHD) [40]	2014	Congenital heart disease	40	12.9 ± 8.1	Same as above	An iE33 system with an X5-1 transducer (Philips Medical Systems) or Vivid E9 with a V4 transducer	same	Automatic
Laser et al. 2014 (Healthy control) [40]	2014	Healthy control	20	16.5 ± 6.7	Modified acquisition technique for RT3DE followed by KBR software analysis, standard five-chamber view	An iE33 system with an X5-1 transducer (Philips Medical Systems) or Vivid E9 with a V4 transducer	Ventripoint Medical System (VMS) version 1.2.6804.1278 (Ventripoint Diagnostics, Seattle, WA).	Automatic
Neukamm et al. 2014 [41]	2014	All had Tetralogy of Fallot, post pulmonary valve replacement (PVR) (Adults and children post-PVR with biological valve in Gore-Tex conduit)	30	20 (8–61)	Apical four chamber view	Vivid 7 machine (GE Healthcare, Milwaukee, WI, USA).	VentriPoint Medical System (VMS)	Manual
Knight et al. 2015 (Carcinoid with no Valvopathy) [42]	2015	Carcinoid (No Valvopathy), metastatic carcinoid tumours without cardiac involvement	11	59 ± 10	Apical four-chamber	Single-beat full-volume 3D echocardiographic RV data sets were acquired using the 4Z1c matrix-array transducer (frequency bandwidth, 1.5–3.5 MHz; maximum depth, 30 cm; maximum field of view, 90 90).	same	Semi-automatic
Knight et al. 2015 (Carcinoid Heart Disease) [42]	2015	Carcinoid Heart Disease	20	63 ± 8	Apical four-chamber	Single-beat full-volume 3D echocardiographic RV data sets were acquired using the 4Z1c matrix-array transducer (frequency bandwidth, 1.5–3.5 MHz; maximum depth, 30 cm; maximum field of view, 90 90).	same	Semi-automatic
Knight et al. 2015 (Healthy Volunteers) [42]	2015	Healthy Volunteers	20	50 ± 12	Apical four-chamber	Single-beat full-volume 3D echocardiographic RV data sets were acquired using the 4Z1c matrix-array transducer (frequency bandwidth, 1.5–3.5 MHz; maximum depth, 30 cm; maximum field of view, 90 90).	same	Semi-automatic
Knight et al. 2015 (PH) [42]	2015	Pulmonary hypertension	49	56 ± 13	Apical four-chamber	Single-beat full-volume 3D echocardiographic RV data sets were acquired using the 4Z1c matrix-array transducer (frequency bandwidth, 1.5–3.5 MHz; maximum depth, 30 cm; maximum field of view, 90 90). Acuson Siemens SC2000 cardiac ultrasound system.	Single-beat full-volume 3D echocardiographic RV data sets. Full-volume 3D echocardiographic RV data sets were imported into the on-cart RV Analysis application	Semi-automatic
Li et al. 2015 [43]	2015	Pulmonary hypertension patients (13 patients with chronic thromboembolic PAH, 8 with idiopathic PAH, and 2 with connective tissue diseases with PAH)	23	51.6 ± 14.8	Apical view	an x3-1(iE33, Philips Health care) and a 4Z1C matrix-array transducer (Siemens Acuson SC2000)	TomTec 4D RV-Function software	Manual
Lu et al. 2015 [44]	2015	cardiomyopathy, arrhythmias, CHD	60 had 3DE and 57 had CMR	45 ± 10	Modified apical view	GE Vivid 9	TomTec software	Manual
Medvedofsky et al. 2015 [45]	2015	Cardiomyopathy/Heart failure: 47% of the patients, Pulmonary arterial hypertension: 22% of the patients, congenital heart disease: 6% of the patients	131	53 ± 15	Short-axis views (aimed to test a new approach for volumetric analysis without coronal views by using instead right ventricle–focused three-dimensional acquisition with multiple short-axis views extracted from the same data set)	iE33 imaging system equipped with an S5 transducer (Philips Medical Systems, Andover, MA).	Vendor-specific software (TomTec).	Semi-automatic
Selly et al. 2015 (After surgery) [46]	2015	Repaired Tetralogy of Fallot (rTOF) referred for pulmonary valve replacement, Follow-Up Pre-op and 1-year post-op assessments for all patients	26 patients had MRI, but 12 patients had 3 DE	27 ± 12	Apical four-chamber view	Vivid 7®, 3 V matrix-array transducer (1—4 MHz).	TomTec Imaging Systems	Manual
Selly et al. 2015 (Before surgery) [46]	2015	Same as above	26 patients had MRI, but 12 patients had 3 DE	27 ± 12	Apical four-chamber view	Vivid 7®, 3 V matrix-array transducer (1—4 MHz).	TomTec Imaging Systems	Manual
Muraru et al. 2016 [47]	2016	Ischemic heart disease (43% of the patients), congenital heart disease (21% of the patients), cardiomyopathy (15% of the patients)	47	46 ± 20	Apical long-axis view, and RV short-axis view	Vivid E9 (GE)	TomTec Imaging Systems	Semi-automatic
Park et al. 2016 [48]	2016	44 patients: had severe TR, Atrial septal defect, Atrial fibrillation, and 15 normal subjects	59	46.9 ± 19.3	Modified apical window	single-beat 3D echocardiographic, using Siemens Acuson SC2000 echocardiographic system, a 4Z1c Instantaneous Full Volume transducer (1.5–3.5 MHz).	data were analysed using the Right Ventricular Analysis Application (SC2000 workplace version 1.5; Siemens AG). Vendor-specific.	Semi-automatic
Ishizu et al. 2017 [49]	2017	Underlying Heart Disease: Non-congenital (e.g., cardiomyopathy) 52%, Congenital (e.g., TOF) 48%	75	40 ± 18	Five cross-sectional images were obtained: a four-chamber RV inlet-to-apex view, coronal apex-to-outflow view, and three axial views for the tricuspid annular plane, mid-RV level, and apical level	ARTIDA ultrasound system (Toshiba Medical Systems)	analysed using prototype software for dedicated RV assessment (Toshiba Medical Systems).	Manual
Medvedofsky et al. 2017 [7]	2017	Patients with a wide range of RV size and function (dilated cardiomyopathy (73%), HTN (37%), PAH (20%), Simple congenital heart disease 10%, ESRD 3%)	30	67 ± 16	Apical two and four-chamber views, LV outflow tract diameter in the apical three-chamber view	iE33 or EPIC imaging systems equipped with an S5 transducer (Philips Healthcare, Andover, MA).	4D RV-Function 2.0, a module of TomTec-Arena; TomTec Imaging Systems	Semi-automatic
Nagata et al. 2017 [8]	2017	60 patients who received a clinically indicated CMR examination and agreed to undergo a 3DTTE examination on the same day were prospectively enrolled.	60	61 ± 15	Apical 4- and 2-chamber views	An iE33 or Epic 7 G scanner (Philips Medical Systems) equipped with a fully sampled matrix-array transducer (X3 or X5-1)	4D LV Analysis, version 3.1.2 and 4D RV Function 2.0; TomTec Imaging Systems	Semi-automatic
Aubert et al. 2018 (no PH) [50]	2018	Healthy subjects	11	58.8 ± 10.5	Apical four- and two-chamber views, apical three-chamber view	V9 and V95 systems with the M5S probe (GE Vingmed Ultrasound, Horten, Norway)	TomTec software (4D RV-Function 2.0, TomTec-Arena; TomTec Imaging Systems, Unterschleissheim, Germany).	Semi-automatic
Aubert et al. 2018 (Patients with PH) [50]	2018	Patients with pulmonary hypertension	79	58.1 ± 14.9	Apical four- and two-chamber views, apical three-chamber view	V9 and V95 systems with the M5S probe (GE Vingmed Ultrasound, Horten, Norway)	TomTec software (4D RV-Function 2.0, TomTec-Arena; TomTec Imaging Systems, Unterschleissheim, Germany).	Semi-automatic
Genovese et al. 2019 [51]	2019	Normal (31% of the patients), HFrEF (21% of the patients), IHD (14% of the patients)	56	52 ± 16	Apical four-chamber (4Ch) RV-focused view	An iE33 or EPIQ 7C system (Philips Healthcare, Andover, MA), equipped with an X5-1 phase array transducer. Used machine learning (ML)-based 3D echocardiography (3DE) algorithm	New Machine Learning approach (3D Auto RV, Philips Healthcare) on a commercial platform for data management (QLAB, Philips)	Fully automated analysis was accurate in 32% of patients, Minimal manual editing was required in 68% of patients
Greiner et al. 2019 [52]	2019	Normal myocardial function without significant CAD:15 patients, CAD: 10 patients, Cardiomyopathy: 14 patients, Cardiac arrhythmia: 4, Post heart transplantation: 2, Atrial septal defect: 1, Systemic vasculitis:1	42	53 ± 19	Apical 4 chamber window	Vivid E9 BT 11, GE Healthcare Vingmed, Trondheim, Norway. using a 1.5e4.6 MHz phased array probe (M5S-D) and an active-matrix four-dimensional volume phased array probe (4 V-D).	EchoPAC workstation BT11, GE Healthcare. 3D RV volumetry done by 4D-RV function, TomTec Imaging Systems that was embedded in the EchoPAC workstation	Semi-automatic
Tamborini et al. 2019 [53]	2019	Normal subjects: 35 participants, Pathological patients: 98 participants (Valvular heart disease: 26 cases, CAD: 20 cases, Idiopathic dilated cardiomyopathy: 33 cases, Congenital or acquired pathologies with RV pressure/volume overload: 26 cases	133 had 3 De and 22 CMR	63 ± 17	4-chamber apical view	GE vivid E95 with M5Sc-D probe (2D) and 4 V-D probe (3D)	On-board (OB): 4D RV-Function 2.0	Automatic
Otani et al. 2020 (Fully automated) [54]	2020	Ischemic heart disease (36%), Secondary cardiomyopathy (26%), Pulmonary hypertension (7%), Valvular heart disease 9%, Dilated cardiomyopathy 5%	87	67 ± 14	Apical four-chamber view, coronal view, and basal short-axis view	Philips Healthcare	3D Auto RV, Philips Medical Systems (it detects RV endocardial surfaces using artificial intelligence, followed by 3D speckle tracking analysis	Automatic
Otani et al. 2020 (Semi-automated) [54]	2020	Same as above	87	67 ± 14	Apical four-chamber view, coronal view, and basal short-axis view	Philips Healthcare	RV-Function 2 TomTec Imaging Systems GmbH	Semi-automatic
Ahmad et al. 2021 (RV results fully automated) [4]	2021	Normal: 12 subjects, ischemic heart disease: 19 patients, Dilated cardiomyopathy: 53 patients, Valvular heart disease: 15 patients, Hypertrophic cardiomyopathy: 16 patients, Heart transplantation recipients: 18 patients, Hypertensive heart disease: 8 patients, Multiple myeloma: 3 patients, Perinatal cardiomyopathy: 2 of the patients, Uremic cardiomyopathy: 1pateint, Rheumatic heart disease: 1 patient	149	46 ± 15	Apical four chamber, coronal, and basal short-axis views	Philip echocardiographic system	3D auto RV, Philips Healthcare	Automatic
Ahmad et al. 2021 (RV results with manual editing) [4]	2021	Same as above	149	46 ± 15	Apical four chamber, coronal, and basal short-axis views	Philip echocardiographic system	3D auto RV, Philips Healthcare	Semi-automatic
Kamińska et al. 2021 [11]	2021	Arrhythmia (26 patients), Severe ventricular arrhythmia (15 patients), Severe idiopathic ventricular arrhythmia (12 patients), ARVC (3 patients)	41	13.7 ± 3.8	Apical window	Philips EPIQ system	TomTec Imaging Systems	Semi-automatic
Lattanzioa et al. 2021 (Healthy control) [3]	2021	Healthy control	27	41.7 ± 15.7	Complete transthoracic echocardiogram	Vivid E95 (the transducer GE Healthcare 4 V-D Probe Collector)	software GE EchoPAC	Semi-automatic
Lattanzioa et al. 2021 (PAH) [3]	2021	Pulmonary arterial hypertension	34	61.8 ± 15.7	Complete transthoracic echocardiogram	Vivid E95 (the transducer GE Healthcare 4 V-D Probe Collector)	software GE EchoPAC	Semi-automatic
Li et al. 2021 [55]	2021	Dilated cardiomyopathy (51 patients), ischemic heart disease (20 patients), Hypertrophic cardiomyopathy (14 patients), Heart transplantation recipients (21 patients), Heart valvular disease (17 patients), COVID-19 infection (20 patients), Healthy subjects (17 subjects), Others (viral myocarditis, peripartum cardiomyopathy, etc.)	174	47 ± 16	RV-focused apical four-chamber view in full-volume mode with a median FR of 22 (interquartile range, 20–32) frames/sec	3D-STE (3D Speckle-Tracking Echocardiography), performed using Philips echocardiographic systems (EPIQ 7C; S5-1, X5-1 transducer; Philips Healthcare, Andover, MA).	4D RV Analysis, ver. 2.0 (TOMTEC)	Automatic
Myhr et al. 2021 [2]	2021	Healthy subjects: 45 (53%). Cardiac disease patients: 40 (47%): Hypertrophic cardiomyopathy: 16 (19%), Aortic valve stenosis (moderate-severe): 6 (7%), Aortic valve insufficiency (moderate-severe): 6 (7%), ischemic heart disease: 3 (4%), Other cardiac conditions: 8 (9%)	55 had 3 DE, 83 CME	44 ± 14	Parasternal short- and long-axis views to provide images for RVOT measurements as well as a focused four- chamber view of the RV for dimensions.	Vivid E95 (GE Healthcare), 4 V-D volume phased array (1.5–4 MHz), Frame Rate: 21 ± 7 volumes per second	4D RV-Function 2.0 (TomTec Imaging Systems)	Semi-automatic
Namisaki et al. 2021 (4CV Automated) [56]	2021	Secondary cardiomyopathy: 55 patients (32%), IHD: 55 patients (32%), Valvular heart disease: 20 patients (11%), Dilated cardiomyopathy: 13 patients (7%), PHT: 7 patients (4%), Hypertrophic cardiomyopathy: 4 patients (2%), Others: 20 patients (11%)	153	67 ± 14	Apical four-chamber view (4CV)	Epic 7 G with X5-1 transducer (Philips Medical Systems), Acquisition: One-beat acquisition (HMQ) mode	Analysis Software: 3D Auto RV, QLAB version 13 (Philips)	Automatic
Namisaki et al. 2021 (4CV Manual editing) [56]	2021	Same as above	153	67 ± 14	Apical four-chamber view (4CV)	Epic 7 G with X5-1 transducer (Philips Medical Systems), Acquisition: One-beat acquisition (HMQ) mode	Analysis Software: 3D Auto RV, QLAB version 13 (Philips)	Semi-automatic
Namisaki et al. 2021 (RVFV Automated) [56]	2021	Same as above	153	67 ± 14	RV-focused view (RVFV)	Epic 7 G with X5-1 transducer (Philips Medical Systems), Acquisition: One-beat acquisition (HMQ) mode	Analysis Software: 3D Auto RV, QLAB version 13 (Philips)	Automatic
Namisaki et al. 2021 (RVFV Manual editing) [56]	2021	Same as above	153	67 ± 14	RV-focused view (RVFV)	Epic 7 G with X5-1 transducer (Philips Medical Systems), Acquisition: One-beat acquisition (HMQ) mode	Analysis Software: 3D Auto RV, QLAB version 13 (Philips)	Semi-automatic
Zhong et al. 2021 [9]	2021	25 children with functional single right ventricle (FSRV) post-Fontan; 25 healthy controls	50	3.7 ± 1.8	Apical four-chamber view	Philips, Andover, with a matrix-array transducer (X5-1).	TomTec 4D RV analysis software	Automatic
Zhu et al. 2021 [5]	2021	Ischemic heart disease, Hypertrophic cardiomyopathy, Dilated cardiomyopathy, Myocarditis, Normal heart	51	52 (34–59)	apical four-chamber view	EPIQ CVx system (Philips Healthcare, Andover), an X5-1 transducer	AI-based 3DE software,	Automatic
Mathijssen et al. 2022 [57]	2022	Pulmonary sarcoidosis patients	122	50.9 ± 12.0	A series of standard and nonstandard TTE views were obtained: parasternal long and short axis, standard apical 4-chamber and focused RV apical.	iE33 system and S5 transducer Philips Medical Systems	TTE-KBR (knowledge-based reconstruction) images were made according to the VentriPoint user guide (VentriPoint Diagnostics Ltd. Seattle, USA)	Automatic
Colak et al. 2023 [10]	2023	Heart transplant recipients	35	32 ± 5	RV-focused apical four-chamber view	GE Vivid E9	EchoPac software	Manual
De Bosscher et al. 2023 [58]	2023	201 elite endurance athletes	201	18 (17–20)	2 apical views (4-chamber and RV 4-chamber orthogonal) and two short-axis (apical and basal)	Vivid E9 or Vivid E95, GE Healthcare, 1.5–4 MHz matrix-array transducer (GE 4Vc-D Matrix 4D cardiac probe)	Echo PAC version 203, GE Healthcare	automatic
Vitarelli et al. 2023 [59]	2023	Repaired TOF Patients	24	38.1 ± 11.4	Modified apical 4 chamber	A matrix-array 3D transducer was used, (Vivid E9/E95, GE)	TomTec Imaging Systems used for offline analysis of RV volumes., and Quantification of 3D-strain was performed using GE- EchoPac software version-R5	automatic
Hadeed et al. 2024 [12]	2024	Congenital heart disease (Repaired tetralogy of Fallot: 50 patients. Atrial septal defect + partial anomalous pulmonary venous return: 35 patients. Operated pulmonary valve stenosis/atresia: 10 patients. Repaired truncus arteriosus: 6 patients. Repaired malposition of great arteries with pulmonary valve stenosis: 5 patients)	106 had 3 DE and 27 had CMR	9.3 (IQR 6.2)	Apical four-chamber view, **one-beat full-volume mode**	Vivid E95 (GE Healthcare) with 4VC-D probe	RV Quantification (RVQ) for semiautomatic contouring	Semi-automatic
Pozza et al. 2024 [60]	2024	Congenitally corrected transposition of great arteries (cc-TGA): 11 patients (57.9%), Dextro-transposition of great arteries (D-TGA): 8 patients (42.1%)	11 had 3 DE and 19 CMR	28 (17.25–33)	Apical 4 chamber window	Vivid E95 ultrasound system	Echo PAC version 112.99, Research Release, GE Healthcare	Semi-automated
Yanagi et al. 2024 [61]	2024	Patients with atrial septal defect (ASD) undergoing transcatheter closure	83	50 ± 18	Apical 4 chamber	3D Auto RV, QLAB version 15.0, Philips Healthcare; median frame rate, 22 Hz	1- beat acquisition mode (Dynamic Heart Model, QLAB version 15.0, Philips Healthcare;	Automatic (but manual editing was used when needed)

**Abbreviations:**

3DTTE: 3D transthoracic echocardiography

4CV: apical four chamber view

ABD: Automated Border Detection

AR: Apical rotation method

ARVC: arrhythmogenic RV dysplasia

CAD: coronary artery disease

CHD: congenital heart disease

CoA: coarctation of aorta

DCM: dilated cardiomyopathy

DS: Disc Summation

ESRD: end stage renal disease

HFrEF: heart failure with reduced EF

HTN: hypertension

IHD: ischemic heart disease

MABD: Manual Adjusted Border Detection

PAH: pulmonary arterial hypertension

PH: pulmonary hypertension

PHT: pulmonary hypertension

RVFV: RV focused view

STEMI: ST segment elevation myocardial infarction

TGA: transposition of great arteries

TOF: tetralogy of Fallot

VSD: ventricular septal defect

**Fig. 2:**
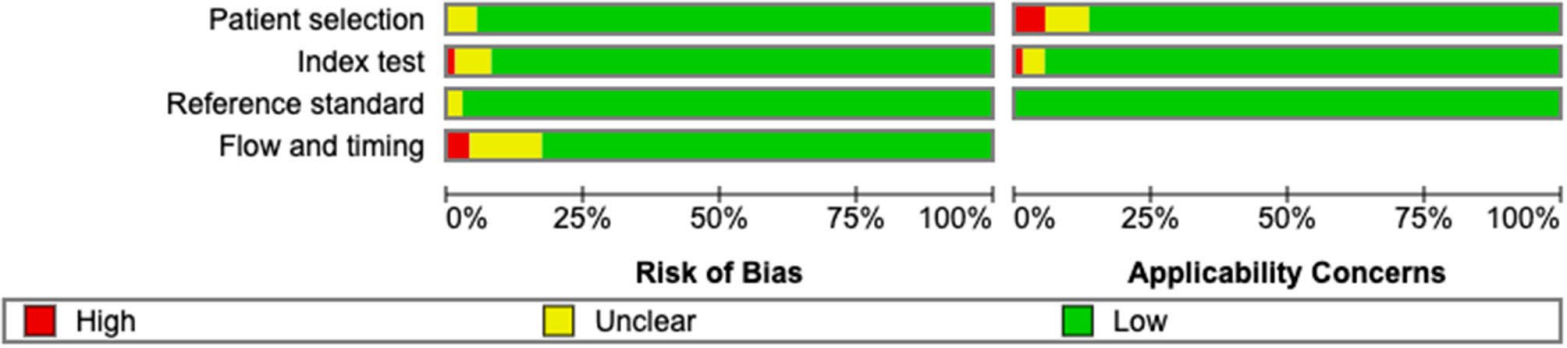
Methodological_quality graph

**Fig. 3:**
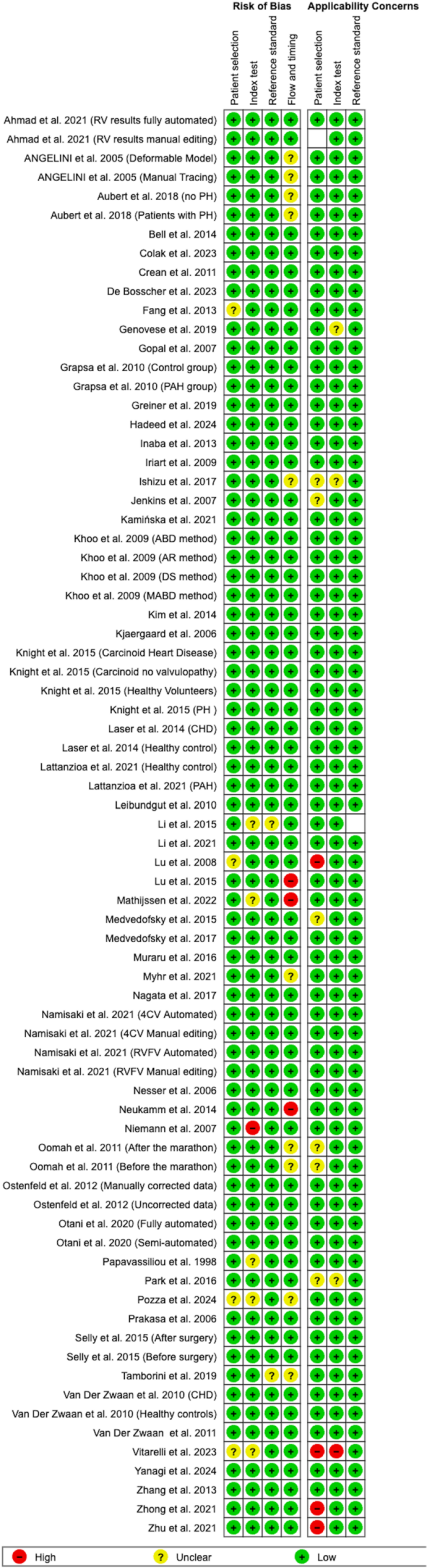
Methodological_quality summary

Quality assessment using QUADAS-2 revealed that the majority of the studies demonstrated good methodological quality with low risk of bias across all domains and minimal applicability concerns (Figs. 2 and 3).

### Meta-analysis of systematic bias

**Fig. 4:**
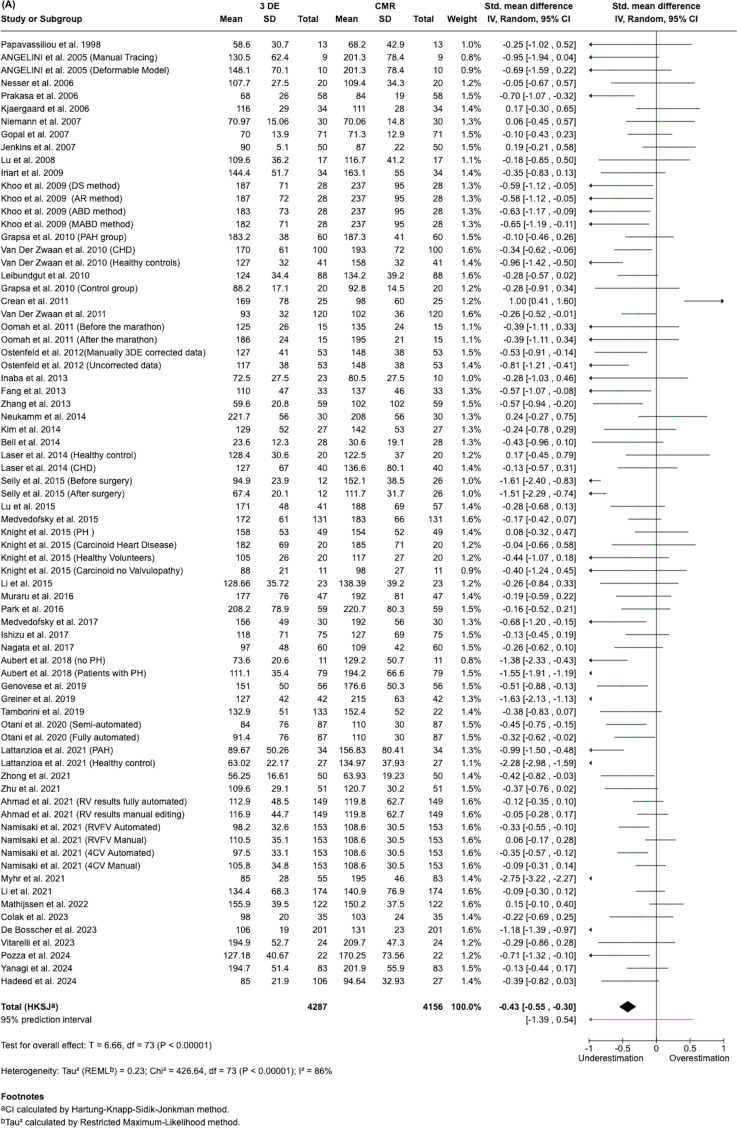

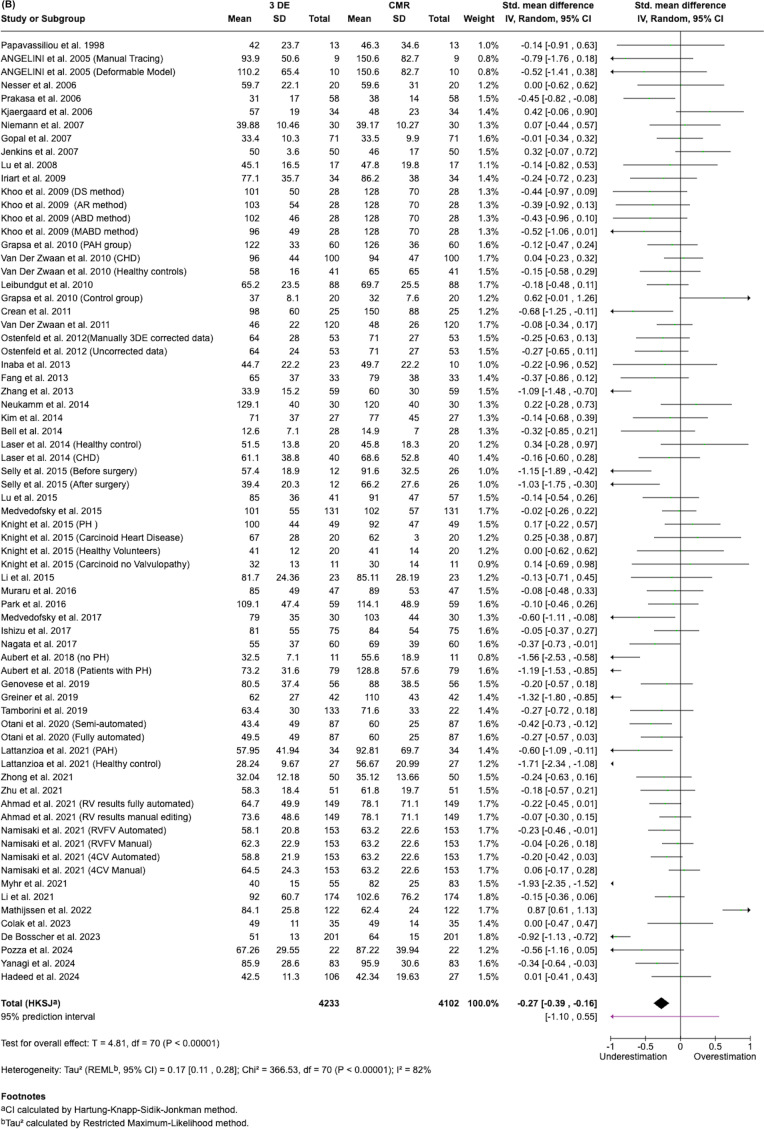

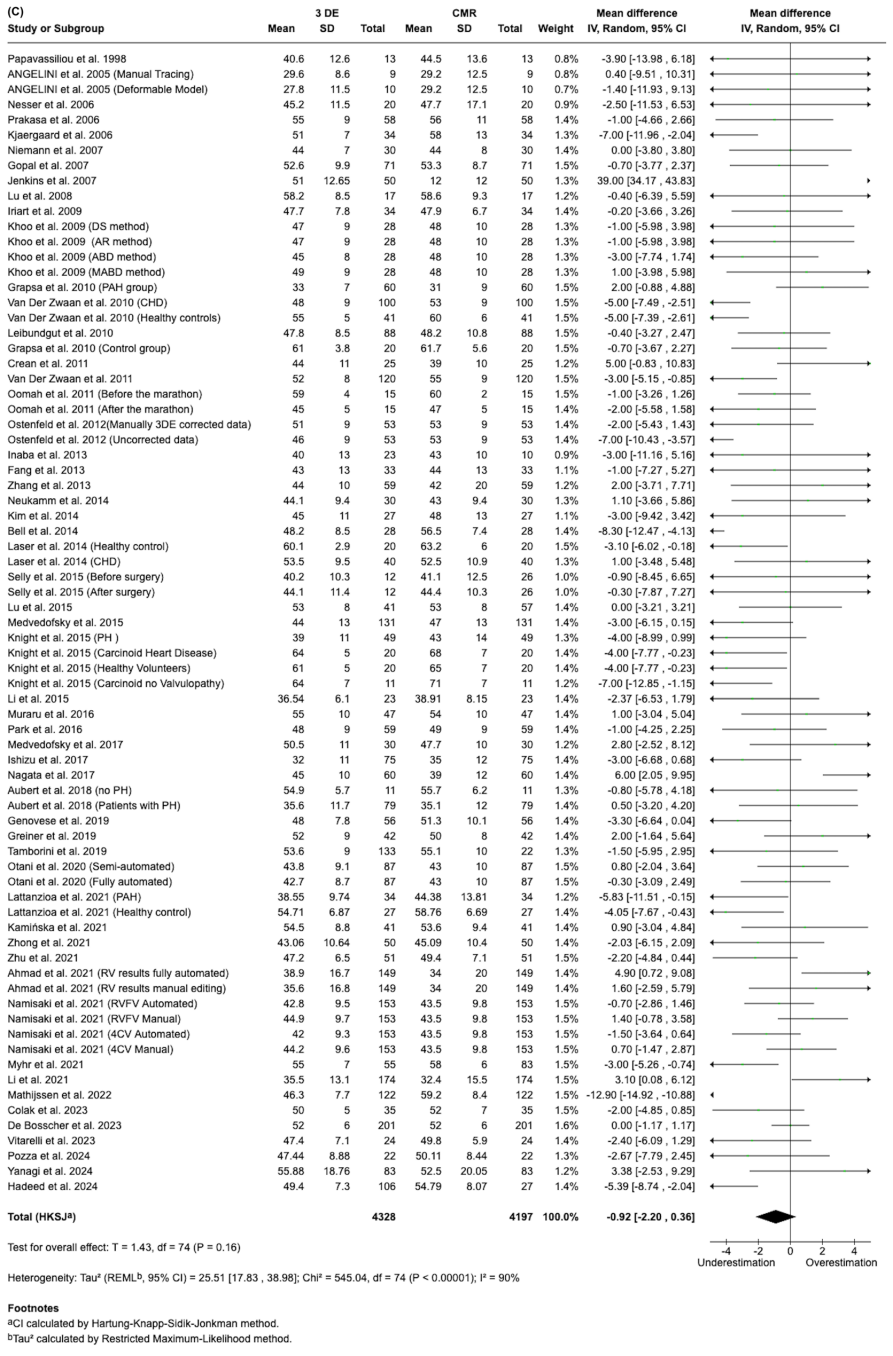
Forest plots summarizing pooled estimates of EDV ESV, and EF across studies (top to bottom)

In this meta-analysis, we compared 3DE to CMR across three cardiac parameters: Ejection Fraction (EF), End Systolic Volume (ESV) and End Diastolic Volume (EDV). Approximately 4300 patients were included across 71 to 75 studies, depending on the parameter assessed (Fig. 4).

Our analysis showed that 3DE consistently underestimated ESV and EDV compared to CMR, with statistically significant differences (*p* ≤ 0.00001). The greatest underestimation was observed for EDV (SMD = −0.43, 95% CI: −0.55, −0.30: 74 studies), followed by ESV (SMD = −0.27, 95% CI: −0.39, −0.16: 71 studies). For EF, 3DE showed a minimal underestimation compared to CMR (mean difference = −0.92%, 95% CI: −2.20 to 0.36: 75 studies), but this difference was not statistically significant (*p* = 0.16), indicating good agreement between the two methods for EF assessment. However, substantial heterogeneity was observed across all 3 parameters (I^2^ = 82–90%), with EF showing the highest heterogeneity (I^2^ = 90%).

### Correlation analysis

The pooled values of the correlation coefficients showed a strong positive correlation between 3DE and CMR for all parameters: EDV (0.88, 95% CI: 0.86–0.91; 61 studies), ESV (0.89, 95% CI: 0.87–0.91; 60 studies), and EF (0.79, 95% CI: 0.76–0.82; 69 studies), all of which showed statistically significant heterogeneity (I^2^ : 92.7%, 90.7%, and 91.1% respectively), Supplementary Fig. 5.

### Subgroup analysis

We conducted subgroup analysis to explore sources of heterogeneity, comparing participants below and over 18 years of age, endocardial tracking method, cardiac function status/heart structure as well as indexed vs. non indexed volumes. Both SMD and MD were calculated for statistical pooling and clinical interpretation, respectively.

Subgroup analysis by age did not show a statistically significant difference between the paediatric population (9 studies) and adults (62 studies) for 3DE and CMR regarding EF assessment with an MD of −2.21% (*p* = 0.07) and −0.73% (*p* = 0.31), respectively. For EVD and ESV, paediatric population showed smaller bias but significant subgroup differences (*p* = 0.02 and 0.04, respectively), Supplementary Fig. 6.

For endocardial tracking, semi-automatic tracking provided the closest agreement between modalities for EF assessment, with confidence intervals crossing zero. Manual tracking demonstrated the most consistent results for EF assessment (I^2^ = 0%), while automatic tracking showed the smallest bias for volume parameters (EVD and ESV), Supplementary Fig. 7.

Analysing patients by cardiac function status demonstrated that patients with normal hearts showed the largest underestimation for EF (mean difference = −2.12%, *p* = 0.0008), while patients with diseased hearts demonstrated better agreement between modalities, Supplementary Fig. 8.

Finally, subgroup analysis by volume indexation showed underestimation for both indexed EDV and ESV (SMD: −0.43 and −0.27 respectively), and non-indexed EDV and ESV (MD: −18.56 ml and −8.14 ml respectively). High heterogeneity persisted in both subgroups (I^2^ = 87–94%).

### Additional analysis

Meta-regression analysis of potential moderators (mean age, sample size, and year of publication) for EF showed no associations and failed to explain the observed heterogeneity (omnibus *p* values: 0.465, 0.058 and 0.812 respectively).

Sensitivity analyses was performed by systematic removal of studies; however, it did not significantly reduce heterogeneity (I^2^ remained > 80%).

Moreover, funnel plot inspection and Egger’s tests (RVEDV (*p* = 0.304), RVESV (*p* = 0.68), and RVEF (*p* = 0.67)) indicated minimal publication bias across all parameters.

## Discussion

This meta-analysis represents the largest comparative study addressing the long-standing question of whether 3DE could replace CMR as the preferred modality of measuring RV function. We included 75 studies with approximately 4300 participants.

### Main findings

Our meta-analysis demonstrated that despite consistent underestimation of RV EDV and ESV by 3DE compared to CMR (SMD = −0.43 and −0.27, respectively), it showed better agreement between both modalities for EF with minimal and statistically non-significant bias (MD = −0.92%, *p* = 0.16). It also showed strong correlations between 3DE and CMR for the three parameters (pooled values of the correlation coefficients: 0.88, 89 and 0.79 for ED, ESV and EF, respectively. These robust correlations support the potential use of 3DE for monitoring and comparative assessments in clinical practice.

### Factors affecting measurement accuracy

**Semi-automatic** endocardial tracking provided superior accuracy for EF assessment, with confidence intervals crossing zero, indicating no significant systematic bias. This may be explained by the human input, which can amend or correct algorithmic errors in case of complex anatomy or suboptimal imaging.

In addition, subgroup analysis by **age** revealed that paediatric patients demonstrated smaller measurement bias compared to adults, likely reflecting better acoustic windows, reduced lung interference, and simpler cardiac geometry in younger patients. These findings suggest clinical utility for 3DE in paediatric populations, including congenital heart disease monitoring.

Moreover, patients with **cardiac disease** demonstrated better agreement for all parameters (EDV, ESV and EF), which is likely due to larger chamber size and more distinct landmarks in diseased hearts that helped with more accurate 3DE measurements.

### Comparison with previous literature

Our findings align with the meta-analysis done by Shimada et al. [15], which demonstrated systematic RV volume underestimation by 3DE in 23 studies with 807 subjects. However, our analysis highlights important advances; while Shimada et al. found significant EF underestimation (*p* = 0.03), our analysis demonstrates non-significant EF bias, suggesting meaningful improvements in EF assessment accuracy. Although systematic underestimation of RV volumes persists despite 15 years of technological advances, the improvement in EF agreement suggests the potential clinical utility of 3DE for functional cardiac assessment.

Our work also extends the meta-analysis by Kitano et al. (2023) [62], who found that 3DE underestimated RVEDV by ~ 26 ml, RVESV by~ 10.8 ml, and RVEF by 1.5% relative to CMR (*r* = 0.77–0.89) in 48 datasets, ~2,800 patients. By including newer studies (75 studies/~4,300 patients), and conducting broader subgroup analyses to address heterogeneity, we observed consistent volume underestimation, though of smaller magnitude (MD for non-indexed EDV and ESV was −18.56 ml and −8.14 ml, respectively). In contrast, EF bias was insignificant (MD = −0.92%, *p* = 0.16), suggesting that technological advances have enhanced functional agreement over time. Collectively, our findings support previous evidence while providing greater insights into factors influencing measurement variability such as age, tracking method and patient subgroups, and potential sources of systematic bias.

### Heterogeneity across studies

Substantial heterogeneity persisted across most comparisons (I^2^ = 82–90%) even after conducting extensive subgroup and sensitivity analyses. Meta-regression analysis of available study-level moderators (mean age, sample size, year of publication) showed no significant associations, indicating that these variables do not account for the observed variability. However, this negative finding likely reflects the limitations of our available data rather than absence of true moderators. In addition, the factors most likely to influence 3DE accuracy (such as vendor-specific software algorithms, acquisition protocols, operator experience and image quality metrics) were either inconsistently reported or widely varied across studies, precluding systematic quantitative assessment. Taken together, the substantial heterogeneity observed in our analysis likely represents real and meaningful technical and methodological differences between studies rather than random variation.

Despite this heterogeneity, two key findings remained consistent across analyses: (1) the direction of bias was uniform (volume underestimation), and (2) RVEF showed minimal bias across all subgroups. This consistency in pattern, despite variation in magnitude, suggests that these findings are robust.

Publication bias assessment using Egger’s test showed no significant asymmetry for any parameter (all *p* > 0.304), indicating that our findings are unlikely to be influenced by selective publication of positive results.

### Limitations

Our analysis included studies that used a wide range of 3D echocardiography devices and software over more than two decades. The included studies differed markedly in methodological approaches, which may have introduced technical heterogeneity that could not be accounted for. In addition, there was a high degree of intersubject variability, encompassing patients with diverse cardiac pathologies and healthy volunteers, which may have affected measurement accuracies and limited the generalizability of pooled estimates to specific clinical subgroups. Finally, although 75 studies were included, they represented about 4300 participants spread across many small samples rather than large, consistent cohorts, which may introduce some uncertainty in the pooled results.

## Conclusions

Our meta-analysis demonstrates that 3DE systematically underestimates RV volumes compared with CMR, while showing minimal non-significant bias for EF assessment. In centres with established protocols and experienced operators, 3DE may provide acceptable EF measurements for longitudinal assessment, especially when CMR is not feasible. However, broader clinical adoption requires greater standardisation of image acquisition, analysis, and reporting before it can be considered a routine alternative to CMR.

## Electronic supplementary material

Below is the link to the electronic supplementary material.

Supplementary Material 1. EDV_funnel_plot

Supplementary Material 2. EF_funnel_plot

Supplementary Material 3. ESV_funnel_plot

Supplementary Material 4. ESV_subgroup analysis_indexed vs. non-indexed

Supplementary Material 5. EF_Pooled Correlation Coefficient

Supplementary Material 6. Subgroup analyses by age forest plots

Supplementary Material 7. Forest plots for endocardial tracking subgroups

Supplementary Material 8. Forest plots for subgrouping by heart structure

## Data Availability

The data that support the findings of this study are available from the corresponding author upon request.
