# Supplementary figures and images for: Accuracy of 3-dimensional echocardiography in measuring right ventricular volumes and ejection fraction: a systematic review and meta-analysis
Source: Echo Res Pract. 2026 Jan 29;13:3. doi: 10.1186/s44156-026-00102-w (PMC12853996; doi:10.1186/s44156-026-00102-w)

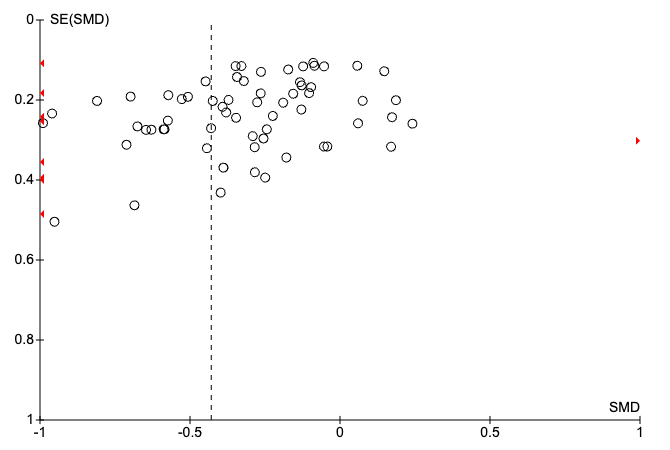

Supplement: Supplementary file 1 — Supplementary Material 1. EDV_funnel_plot [file 44156_2026_102_MOESM1_ESM.png]

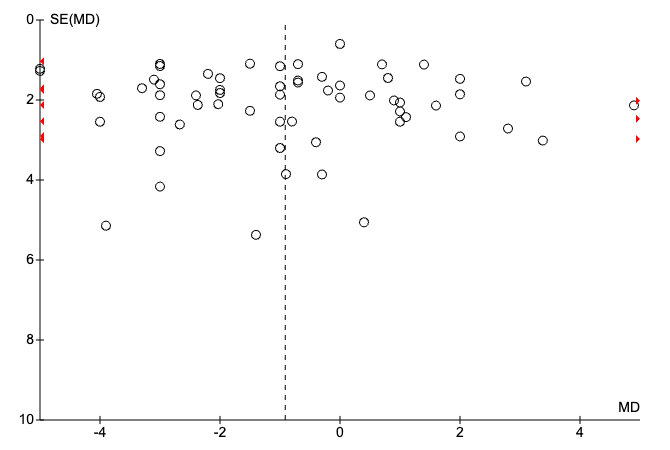

Supplement: Supplementary file 2 — Supplementary Material 2. EF_funnel_plot [file 44156_2026_102_MOESM2_ESM.png]

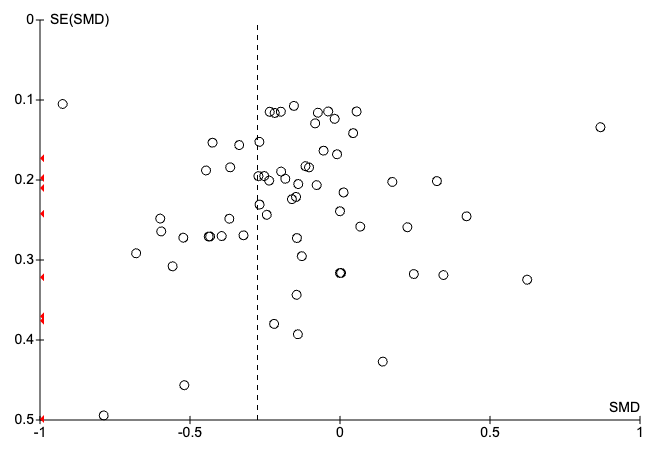

Supplement: Supplementary file 3 — Supplementary Material 3. ESV_funnel_plot [file 44156_2026_102_MOESM3_ESM.png]

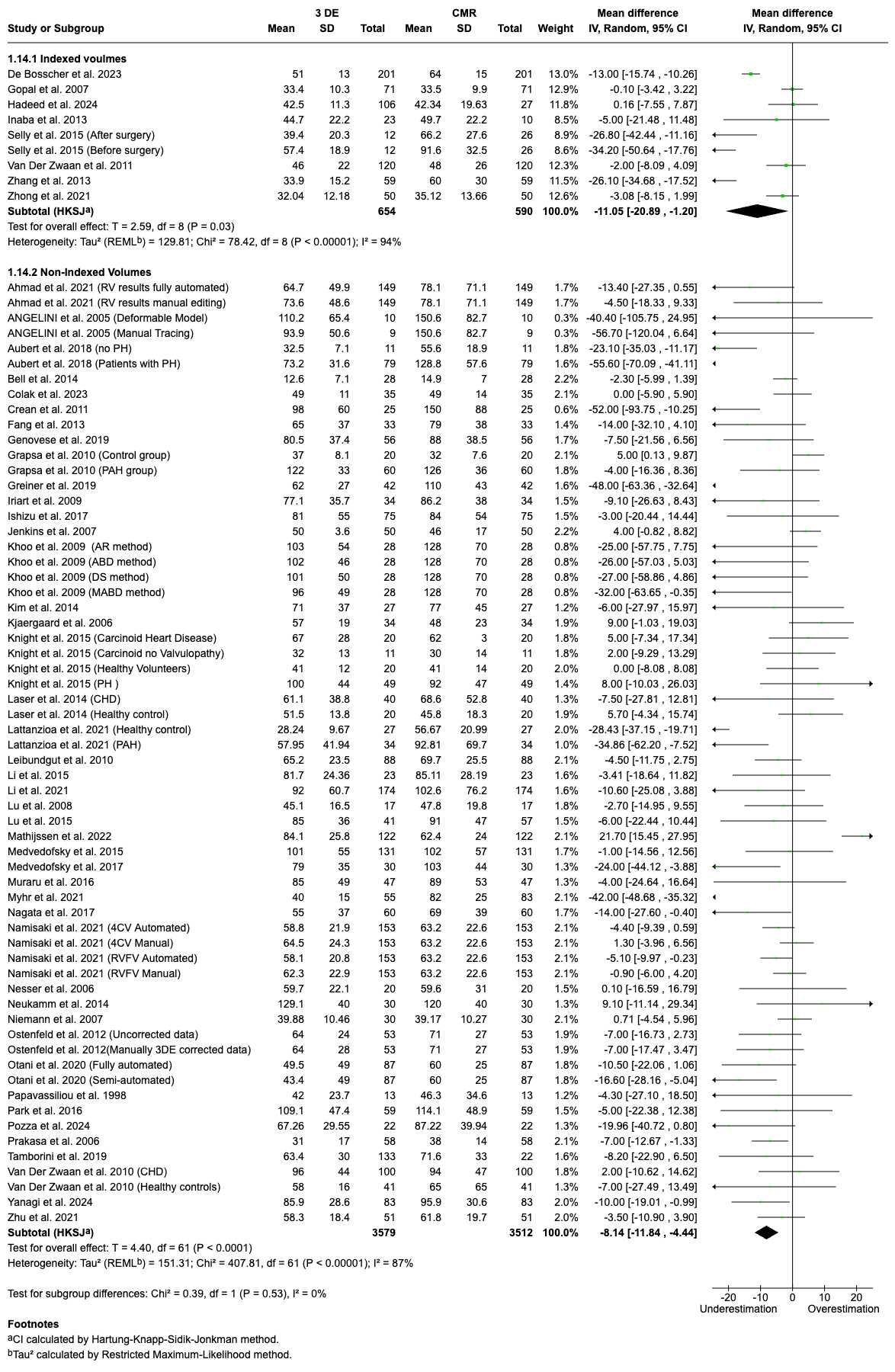

Supplement: Supplementary file 4 — Supplementary Material 4. ESV_subgroup analysis_indexed vs. non-indexed [file 44156_2026_102_MOESM4_ESM.png]

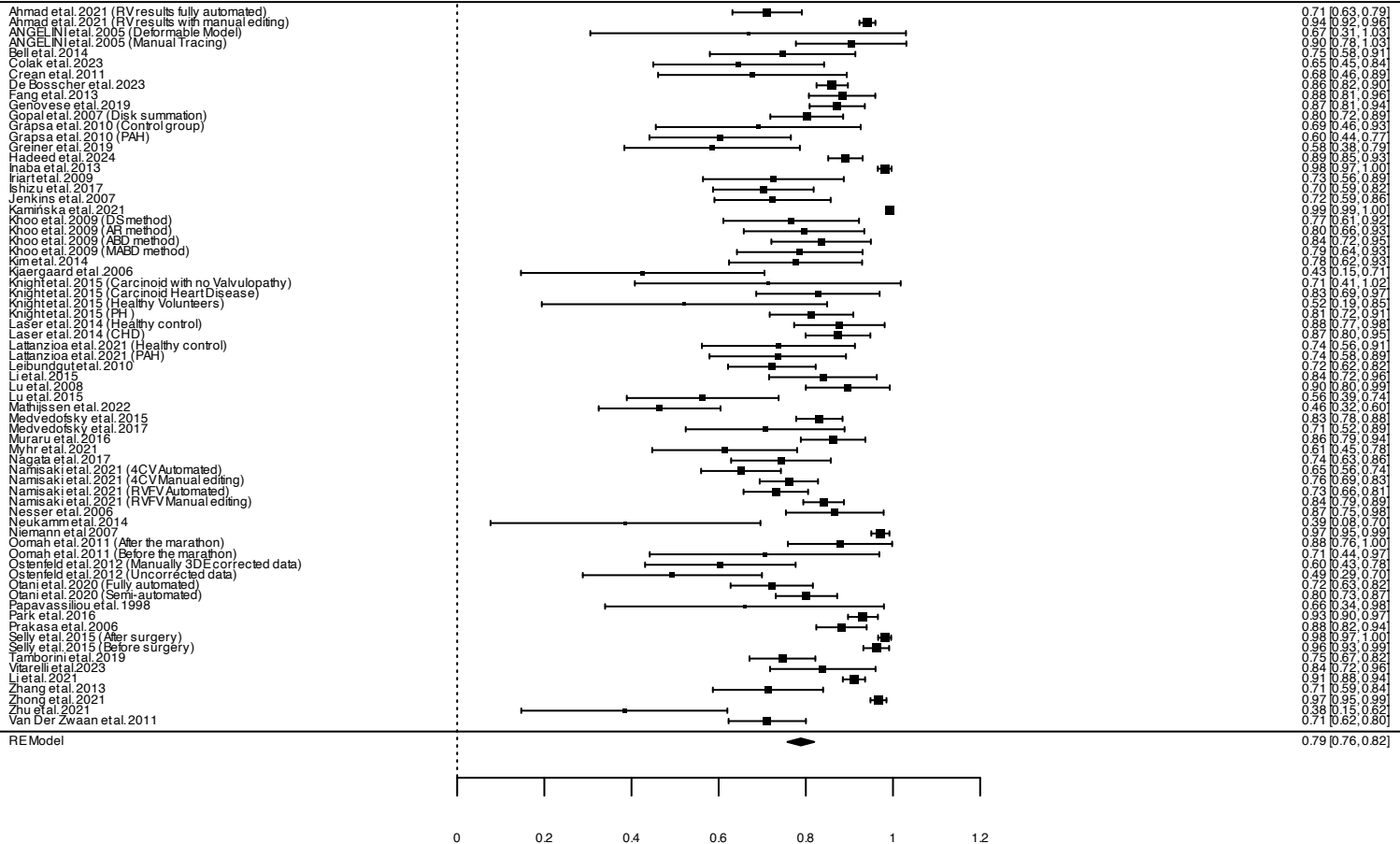

Supplement: Supplementary file 5 — Supplementary Material 5. EF_Pooled Correlation Coefficient [file 44156_2026_102_MOESM5_ESM.pdf]

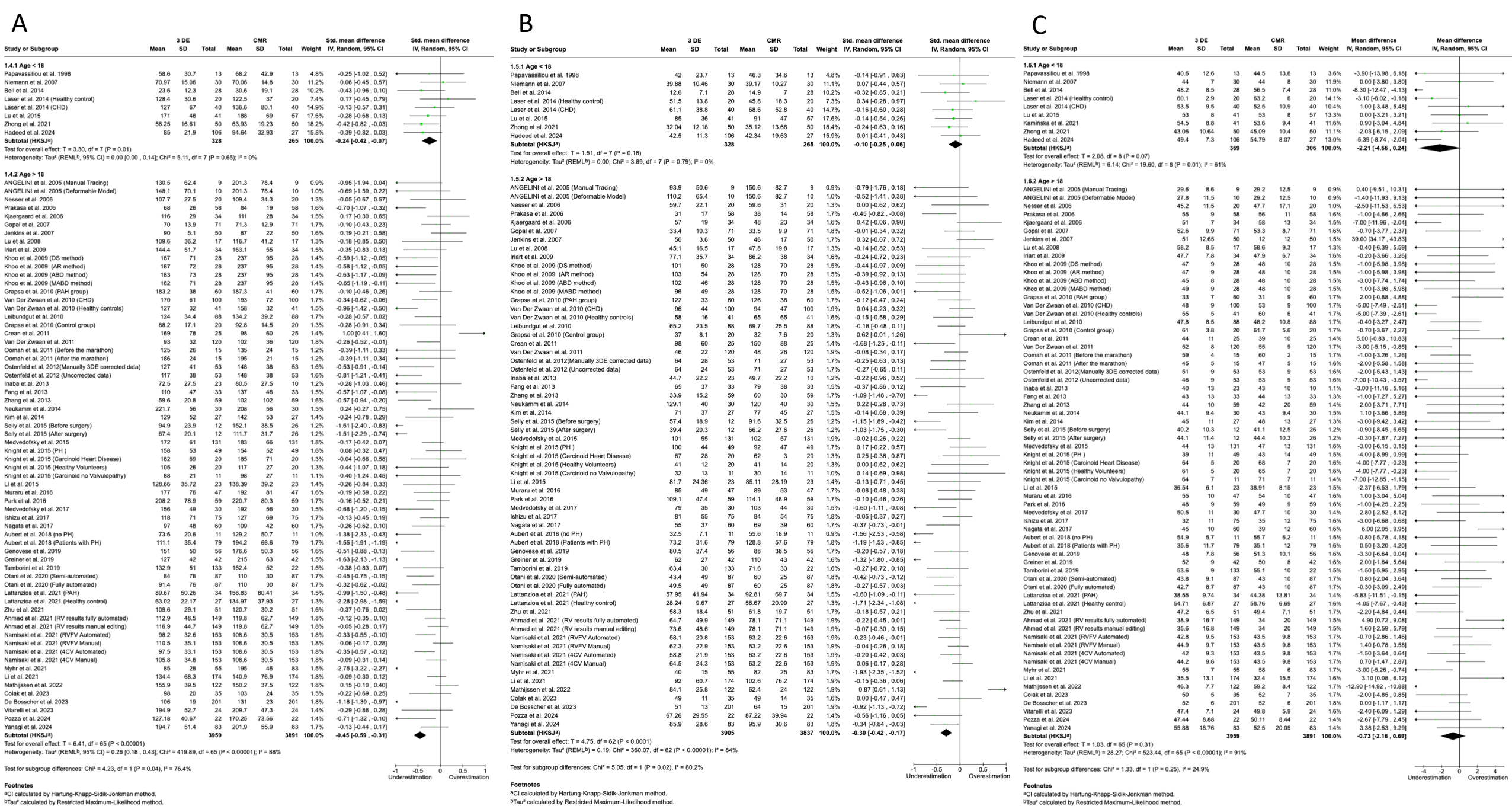

Figure (6): Forest plots showing subgroup analyses stratified by age. EDV (A), ESV (B), EF (C)

Supplement: Supplementary file 6 — Supplementary Material 6. Subgroup analyses by age forest plots [file 44156_2026_102_MOESM6_ESM.pdf]

A

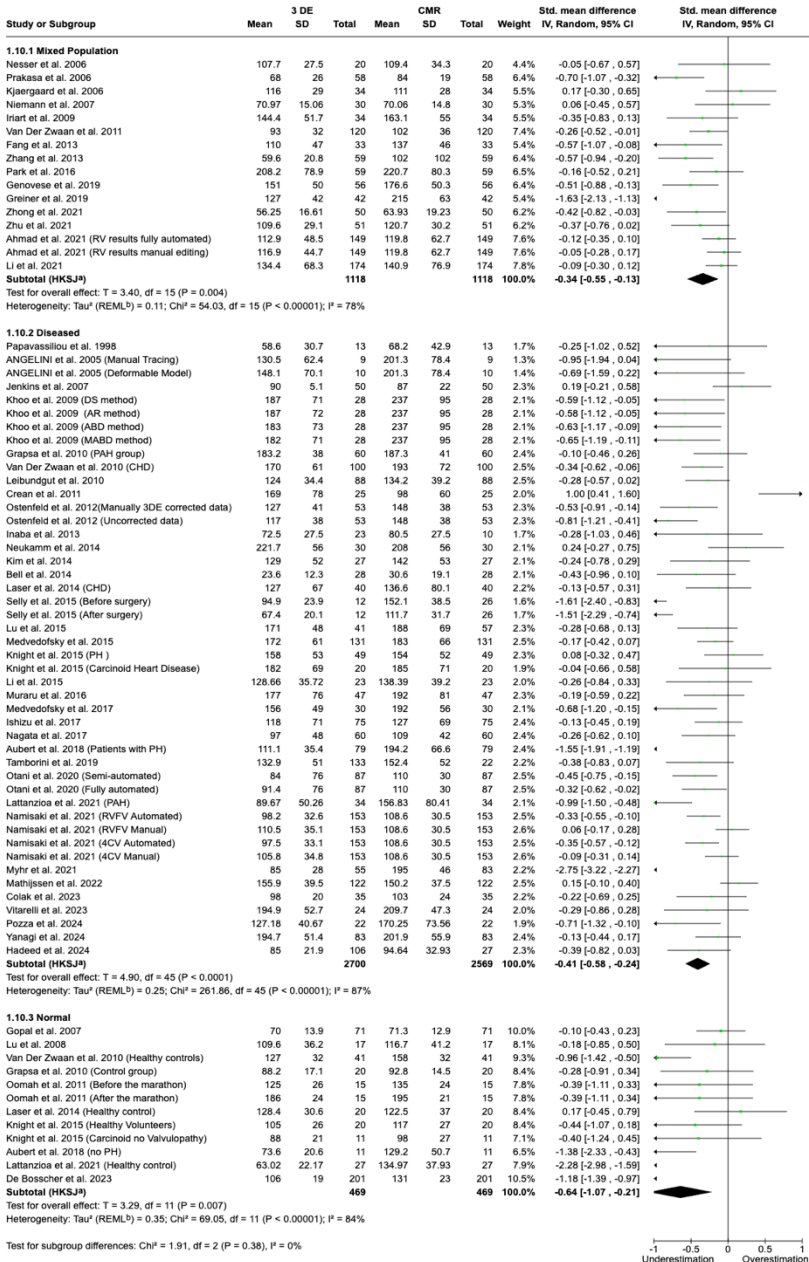

B

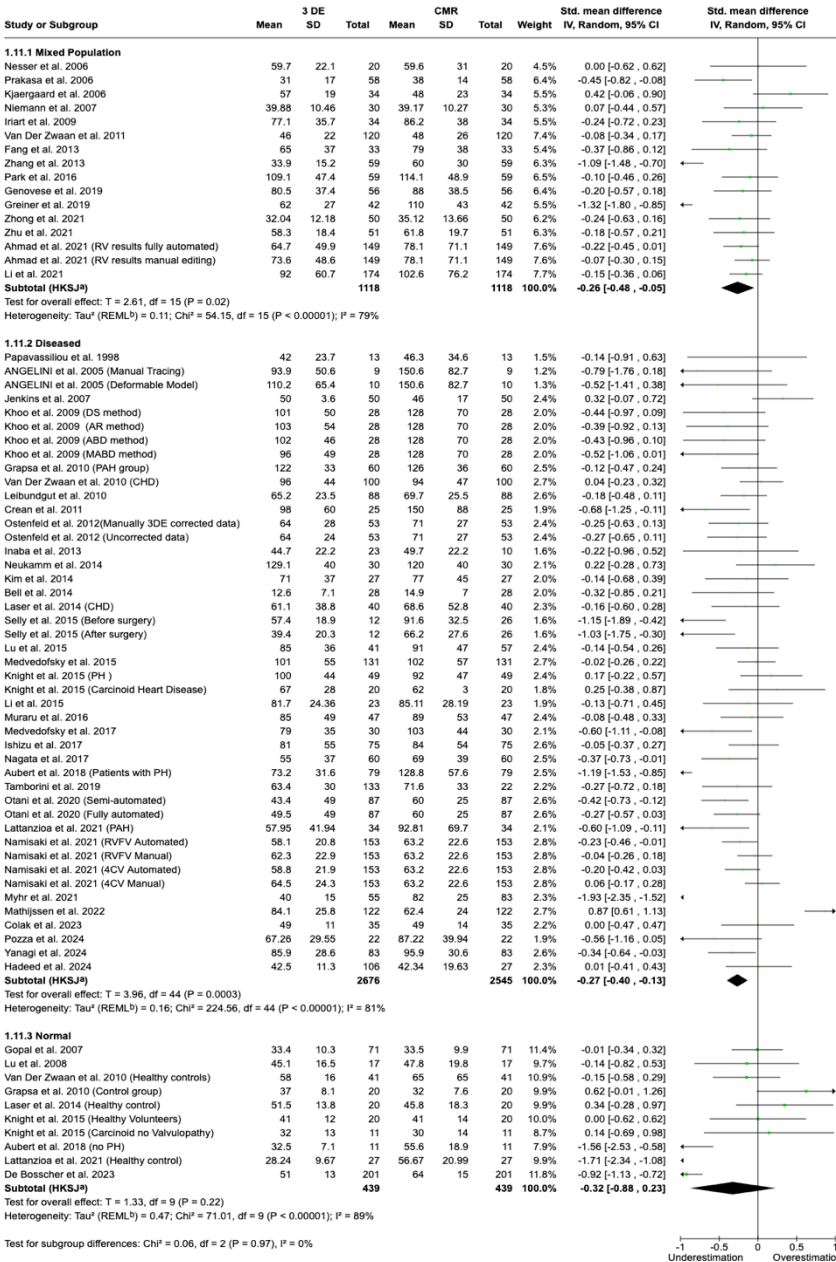

Supplement: Supplementary file 8 — Supplementary Material 8. Forest plots for subgrouping by heart structure [file 44156_2026_102_MOESM8_ESM.pdf]
