# Supplementary material for: Accuracy of 3-dimensional echocardiography in measuring right ventricular volumes and ejection fraction: a systematic review and meta-analysis
Source: Echo Res Pract. 2026 Jan 29;13:3. doi: 10.1186/s44156-026-00102-w (PMC12853996; doi:10.1186/s44156-026-00102-w)
Supplement: Supplementary file 7 — Supplementary Material 7. Forest plots for endocardial tracking subgroups [file 44156_2026_102_MOESM7_ESM.pdf]

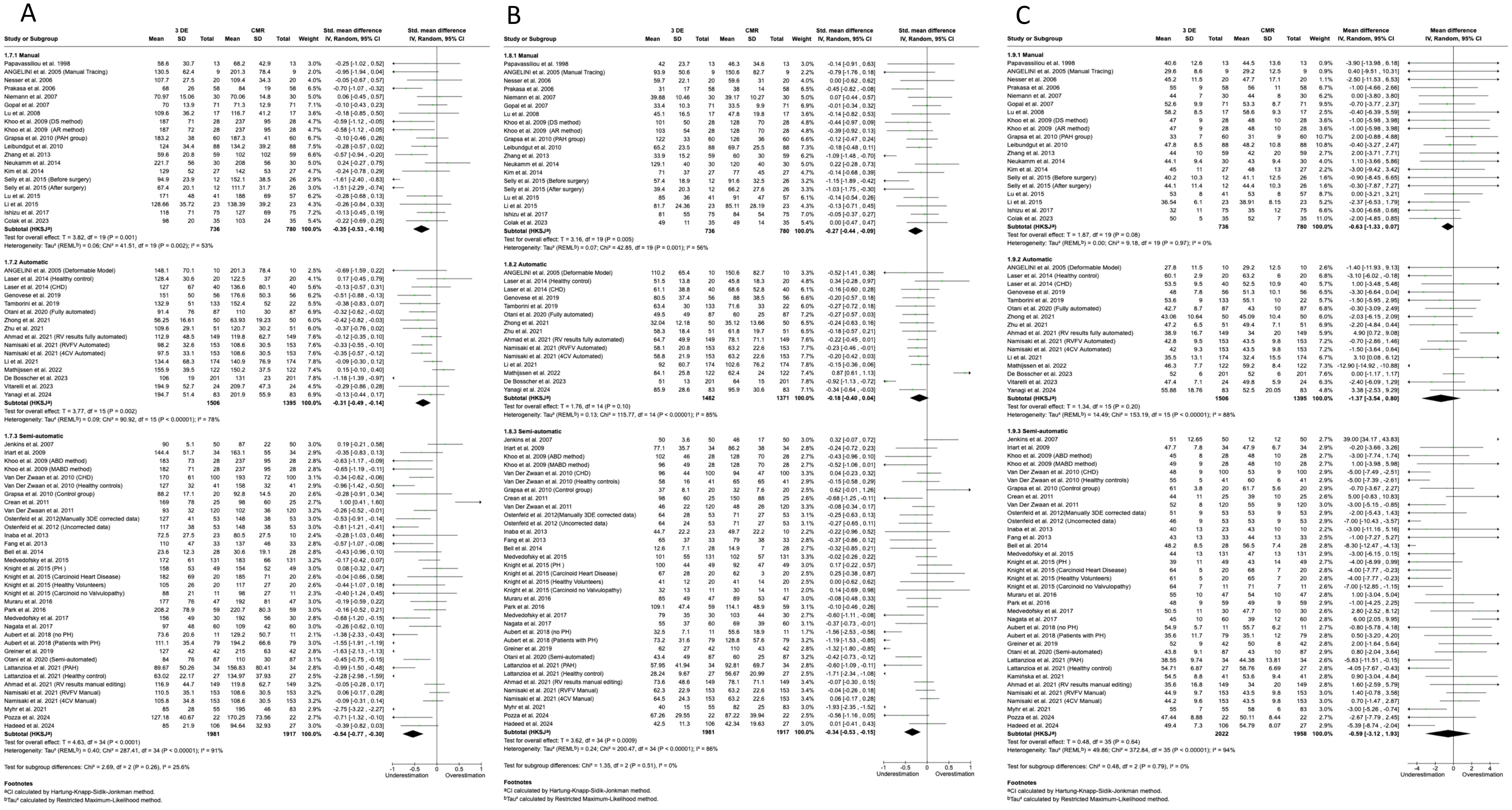

Figure (7) Forest plots showing subgroup analyses stratified by endocardial tracking. EDV (A), ESV (B), EF (C)
